# A Novel Method for Predicting Disease-Associated LncRNA-MiRNA Pairs Based on the Higher-Order Orthogonal Iteration

**DOI:** 10.1155/2019/7614850

**Published:** 2019-05-02

**Authors:** Zhanwei Xuan, Xiang Feng, Jingwen Yu, Pengyao Ping, Haochen Zhao, Xianyou Zhu, Lei Wang

**Affiliations:** ^1^College of Computer Engineering & Applied Mathematics, Changsha University, Changsha 410001, Hunan, China; ^2^Key Laboratory of Intelligent Computing & Information Processing, Xiangtan University, 411105 Xiangtan, China; ^3^Department of Computer Science, Hengyang Normal University, 421008 Hengyang, China

## Abstract

A lot of research studies have shown that many complex human diseases are associated not only with microRNAs (miRNAs) but also with long noncoding RNAs (lncRNAs). However, most of the current existing studies focus on the prediction of disease-related miRNAs or lncRNAs, and to our knowledge, until now, there are few literature studies reported to pay attention to the study of impact of miRNA-lncRNA pairs on diseases, although more and more studies have shown that both lncRNAs and miRNAs play important roles in cell proliferation and differentiation during the recent years. The identification of disease-related genes provides great insight into the underlying pathogenesis of diseases at a system level. In this study, a novel model called PADLMHOOI was proposed to predict potential associations between diseases and lncRNA-miRNA pairs based on the higher-order orthogonal iteration, and in order to evaluate its prediction performance, the global and local LOOCV were implemented, respectively, and simulation results demonstrated that PADLMHOOI could achieve reliable AUCs of 0.9545 and 0.8874 in global and local LOOCV separately. Moreover, case studies further demonstrated the effectiveness of PADLMHOOI to infer unknown disease-related lncRNA-miRNA pairs.

## 1. Introduction

Noncoding RNA, according to its size, can be divided into small and long noncoding RNAs approximately. Generally, small RNAs include tRNAs, miRNAs, piRNAs, and snoRNAs [1–4], and miRNAs are widely present in the cytoplasm of eukaryotic cells and are approximately 18–22 nucleotides in length, which can bind to 3′-untranslated region of mRNA (3′-UTR) to inhibit the translation process of mRNA or to degrade mRNA, thereby affecting the expression of related genes [5–7]. miRNAs play important roles in a series of life activities such as cell differentiation of living body [8], growth and development [9], and apoptosis [10]. Compared to small-molecule ncRNA, lncRNA has a longer nucleotide chain with more than 200 nucleotides and has a specific and complex secondary space structure inside the molecule and can provide multiple sites for protein binding [11]. In addition, both lncRNAs and miRNAs are key members of noncoding RNAs and play important roles in coding and regulation of many complex human diseases [12–16].

Up to now, there have been many studies on relationships between diseases and miRNAs. For example, some important methods proposed by Xing Chen et al. [17–20] and Zou et al. [21–24]. In terms of prediction of potential associations between lncRNAs and diseases, Yu et al. [25] and Xing et al. [26] proposed two kinds of computational models called NBCLAD and LRLSLDA, respectively. Moreover, studies have also shown that there exist relationships between lncRNAs and miRNAs. For example, Gernapudi et al. demonstrated that miRNA 140 can induce the expression of lncRNA NEAT1 [27]. Dey et al. showed that the silencing of lncRNA H19 and knockout of H19 gene in myoblasts significantly decreased skeletal muscle differentiation [28]. Yilong et al. discovered that, after low XIST expression in gliomas, XIST could regulate miR-152 glioma stem cells to inhibit cell proliferation, migration, and invasion [29]. Xinyu et al. demonstrated that lncRNA MALAT1 could achieve posttranscriptional regulation of esophageal squamous cell carcinoma cells through miR-101 and miR-217 [30]. Er-bao et al. proposed that lncRNA ANRIL interacted with miR-99a/miR449a to regulate cell proliferation during gastric cancer formation [31]. You et al. found that the expression of miR-449a and the expression of lncRNA NEAT1 in lung cancer cell L9981 inhibited each other. When miR-449a was overexpressed, NEAT1 expression was decreased, cell proliferation was inhibited, and apoptosis was increased, and vice versa [32]. Emmrich et al. found that the expression of lncRNA MONC and MIR100HG was closely related to the miRNA groups of miR-99a∼125b-2 and miR-100∼125b-1. After silencing of lncRNA MONC and MIR100HG, acute megakaryocytes in the early stage of the disease, the tumor cells of leukemia patients, were severely inhibited [33]. Amy et al. found that lncRNA Ang362 was the host transcriptor of miR-211 and miR-222, and their interactions regulated Ang II and induced proliferation of vascular smooth muscle cells [34]. Miaojun et al. found that the interactions between lncRNA H19 and miRNA-675 play an important role in the metastasis of prostate cancer [35]. Obviously, the exploration of these relationships was conducive to the construction of gene regulatory networks and the identification of the mechanisms of complex human diseases [36–38].

**Figure 1:**
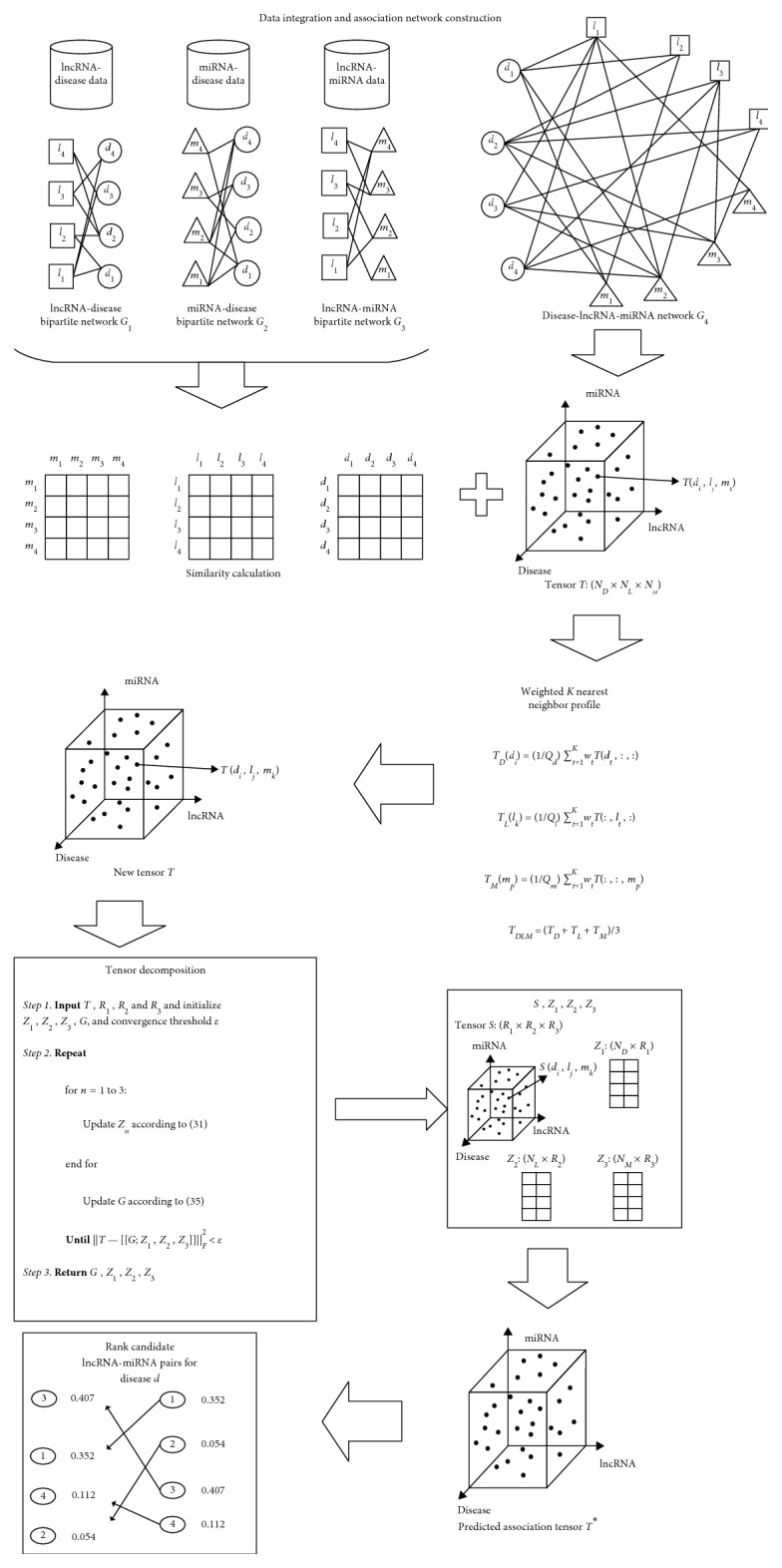
Flow chart of PADLMHOOI for predicting potential associations between diseases and lncRNA-miRNA pairs.

From the above description, it is easy to see that more and more studies have shown that lncRNA-miRNA interactions are involved in the development of complex diseases. However, to the best of our knowledge, so far, in addition to the model of PADLMP proposed by Zhou et al. [39], few models have been proposed for large-scale prediction of potential associations between diseases and lncRNA-miRNA interactions. Hence, inspired by state-of-the-art methods [40–44], which show that the miRNA-miRNA pairs can work cooperatively to regulate a single gene or gene clusters being involved in similar processes [45], and simultaneously, based on the reasonable assumption that functionally similar lncRNA-miRNA pairs tend to be associated with similar diseases, in this paper, a new prediction model called PADLMHOOI was proposed to infer potential associations between diseases and the lncRNA-miRNA pairs. And, as illustrated in Figure 1, our newly proposed prediction model PADLMHOOI consists of the following four major steps:

 
*Step 1 (Data Integration and Network Construction)*. In this step, first of all, we downloaded known disease-lncRNA associations from three different disease-lncRNA databases such as disease-lncRNA [46], MNDR [47, 48], and lnc2cancer [49], respectively, and then, based on these datasets, we constructed a bipartite network of disease-lncRNA. Next, we downloaded known disease-miRNA associations from three different databases such as miR2Disease [50], HMDD [51], and miRCancer [52] separately, and then, based on these datasets, we constructed a bipartite network of disease-miRNA. Moreover, we downloaded the 2015 and 2017 versions of known lncRNA-miRNA associations from the starBasev2.0 database [53] (http://starbase.sysu.edu.cn/) on Feb 2, 2017, and based on these datasets, we constructed a bipartite network of lncRNA-miRNA. Finally, based on these three kinds of bipartite networks, we constructed an integrated tripartite network of disease-lncRNA-miRNA, which could be denoted as a tensor *T*.
 
*Step 2 (Similarity Calculation)*. In this step, we would integrate the disease semantic similarity and Gaussian Interaction Profile Kernel similarity firstly to measure the similarity of diseases. Next, we would integrate the lncRNA functional similarity and miRNA functional similarity in three different ways to measure the functional similarity of lncRNA-miRNA pairs.
 
*Step 3 (Weighted K-Nearest Neighbor Profile)*. Considering that there may be diseases that are unrelated to all lncRNA-miRNA pairs, which may lead to unsatisfactory prediction results while implementing PADLMHOOI to infer potential associations between diseases and lncRNA-miRNA pairs. Hence, in this step, we would introduce the weighted *K*-nearest neighbor profile (WKNNP) to add more interaction information between diseases, lncRNAs, and miRNAs to improve the prediction performance of PADLMHOOI.
 
*Step 4 (Tensor Decomposition)*. In this step, we would perform tensor decomposition on the newly constructed disease-lncRNA-miRNA tensor *T*. Since the results of tensor decomposition include a core tensor and three matrices, we can define the final predicted association tensor as the modal product between the core tensor and these three matrices. Thereafter, we would sort scores of the lncRNA-miRNA pairs associated with each disease in the descending order in the final predicted association tensor, and it is obvious that the higher the ranking of the score, the bigger the possibility that there may exist potential association between the disease and the lncRNA-miRNA pair would be.

## 2. Materials and Methods

### 2.1. Construction of the Bipartite Network of Disease-lncRNA

In order to construct the bipartite network of disease-lncRNA, firstly, known associations between diseases and lncRNAs were downloaded from three different databases such as the LncRNADisease, MNDR, and Lnc2Cancer, respectively, and then, after feature processing (including feature cleaning and data imbalance processing etc.), 2048 different disease-lncRNA associations were finally obtained (Supplementary Table 1). Thereafter, based on these newly obtained 2048 known disease-lncRNA associations, we can construct a disease-lncRNA bipartite network *G*_1_ = (*V*_1_, *E*_1_) according to the following steps:

 
*Step 1*. Let *V*_*l*_1__={*l*_*i*_|*i* ∈ [1, *n*_*l*_1__]} be the set of all different lncRNAs in these 2048 known disease-lncRNA associations and *V*_*d*_1__={*d*_*i*_|*i* ∈ [1, *n*_*d*_1__]} be the set of all different diseases in these 2048 known disease-lncRNA associations, then we define *V*_1_=*V*_*l*_1__ ∪ *V*_*d*_1__ as the vertex set in *G*_1_.
 
*Step 2*. ∀*l*_*i*_ ∈ *V*_*l*_1__, *d*_*j*_ ∈ *V*_*d*_1__, if (*l*_*i*_, *d*_*j*_) belongs to these 2048 downloaded known disease-lncRNA associations, then we define that there is an edge between *l*_*i*_ and *d*_*j*_ in *G*_1_; thereafter, we can obtain the edge set *E*_1_ in *G*_1_.

### 2.2. Construction of the Bipartite Network of Disease-miRNA

In order to construct the bipartite network of disease-miRNA, at first, known disease-miRNA associations were downloaded from three different databases such as the miR2Disease, HMDD, and miRCancer separately, and then, after these newly acquired miRNAs and diseases being mapped to the database miRBase v21 [54] and disease ontology (DO) [55], respectively, 4041 different disease-miRNA associations were finally obtained (Supplementary Table 2). Hence, based on these newly obtained 4041 known disease-miRNA associations, we can construct a disease-miRNA bipartite network *G*_2_ = (*V*_2_, *E*_2_) according to the following steps:

 
*Step 1*. Let *V*_*m*_1__={*m*_*i*_|*i* ∈ [1, *n*_*m*_1__]} be the set of all different miRNAs in these 4041 known disease-miRNA associations and *V*_*d*_2__={*d*_*i*_|*i* ∈ [1, *n*_*d*_2__]} be the set of all different diseases in these 4041 known disease-miRNA associations, then we define *V*_2_=*V*_*m*_1__ ∪ *V*_*d*_2__ as the vertex set in *G*_2_.
 
*Step 2*. ∀*m*_*i*_ ∈ *V*_*m*_1__, *d*_*j*_ ∈ *V*_*d*_2__, if (*m*_*i*_, *d*_*j*_) belongs to these 4041 known disease-miRNA associations, then we define that there is an edge between *m*_*i*_ and *d*_*j*_ in *G*_2_; thereafter, we can obtain the edge set *E*_2_ in *G*_2_.

### 2.3. Construction of the Bipartite Network of lncRNA-miRNA

In order to construct the bipartite network of lncRNA-miRNA, at first, two different versions (2015 and 2017) of lncRNA-miRNA dataset were downloaded from the starBasev2.0 database separately, and then, after feature processing (including feature cleaning and data imbalance processing), 20324 different lncRNA-miRNA interactions were finally obtained (Supplementary Table 3). Thereafter, based on these newly obtained 20324 known lncRNA-miRNA associations, we can construct a lncRNA-miRNA bipartite network *G*_3_ = (*V*_3_, *E*_3_) according to the following steps:

 
*Step 1*. Let *V*_*l*_2__={*l*_*i*_|*i* ∈ [1, *n*_*l*_2__]} denote the set of all different lncRNAs in these 20324 known lncRNA-miRNA associations and *V*_*m*_2__={*m*_*i*_|*i* ∈ [1, *n*_*m*_2__]} denote the set of all different miRNAs in these 20324 known lncRNA-miRNA associations, then we define *V*_3_=*V*_*m*_2__ ∪ *V*_*l*_2__ as the vertex set in *G*_3_.
 
*Step 2*. ∀*l*_*i*_ ∈ *V*_*l*_2__, *m*_*j*_ ∈ *V*_*m*_2__, if (*l*_*i*_, *m*_*j*_) belongs to these 20324 known lncRNA-miRNA associations, then we define that there is an edge between *l*_*i*_ and *m*_*j*_ in *G*_3_; thereafter, we can obtain the edge set *E*_3_ in *G*_3_.

### 2.4. Construction of the Tripartite Network of Disease-lncRNA-miRNA

Based on the above newly obtained networks such as *G*_1_, *G*_2_, and *G*_3_, we can construct a tripartite network *G*_4_ = (*V*_4_, *E*_4_) according to the following steps:

 
*Step 1*. Let *V*_*d*_=*V*_*d*_1__ ∪ *V*_*d*_2__, *V*_*m*_=*V*_*m*_1__ ∪ *V*_*m*_2__, *V*_*l*_=*V*_*l*_1__ ∪ *V*_*l*_2__, *V*_4_={}, *E*_4_={}, and *V*_*d*′_={}.
 
*Step 2*. While *V*_*d*_ is not null, Repeat:
 
∀*d*_*i*_ ∈ *V*_*d*_,
  If ∃*l*_*j*_ ∈ *V*_*l*_ and *m*_*k*_ ∈ *V*_*m*_ satisfyies the following three kinds of conditions simultaneously:

(a) (*d*_*i*_, *l*_*j*_) ∈ *E*_1_
(b) (*d*_*i*_, *m*_*k*_) ∈ *E*_2_
(c) (*l*_*j*_, *m*_*k*_) ∈ *E*_3_

  Then (*d*_*i*_, *l*_*j*_), (*d*_*i*_, *m*_*k*_), and (*l*_*j*_, *m*_*k*_) will be added into *E*_4_ firstly, and then, *d*_*i*_ will be added into *V*_*d*__′_ and removed from *V*_*d*_. Finally, *l*_*j*_ and *m*_*k*_ will be added into *V*_4_ if *l*_*j*_ and *m*_*k*_ are not in*V*_4_.
  Else, *d*_*i*_ will be removed from *V*_*d*_.
 
*Step 3*. Let *V*_4_=*V*_4_+*V*_*d*′_.

According to above steps, a tripartite disease-lncRNA-miRNA association network can be obtained finally. And, it is obvious that, in the tripartite network, there are three kinds of different nodes such as disease nodes, lncRNA nodes, and miRNA nodes; moreover, the number of disease nodes, lncRNA nodes, and miRNA nodes is 68, 44, and 211, respectively, and the number of associations between diseases and lncRNA-miRNA pairs is 3,047.

### 2.5. Construction of the Disease-lncRNA-miRNA Tensor

Based on the newly constructed tripartite network, for any given disease node *d*_*i*_, lncRNA node *l*_*j*_, and miRNA node *m*_*k*_ in *G*_4_, we can define a tensor *T* as follows:

(1) $$\begin{array}{c} \begin{array}{c} T\left(i,j,k\right)=\left\{\begin{array}{cc} 1, & \left[\left(d_{i},l_{j}\right),\left(d_{i},m_{k}\right),\left(l_{j},m_{k}\right)\in E_{4},\right],\\ 0, & \text{otherwise}. \end{array}\right. \end{array} \end{array}$$

### 2.6. Calculation of the Similarity of Disease Pairs

#### 2.6.1. Calculation of the Disease Semantic Similarity (DisSemSim)

In order to estimate the semantic similarity between diseases, we first downloaded the MeSH descriptor from the National Medical Library (http://www.nlm.nih.gov/) and selected the standard MeSH disease terminology. And then, for each disease *d*, we can construct a Directed Acyclic Graph (DAG) such as DAG(*d*)=(*T*(*d*), *E*(*d*)), where *T*(*d*) denotes the set of nodes containing the node *d* itself and its ancestors and *E*(*d*) denotes the set of edges of the respective direct links from parent to child nodes [56]. Thereafter, based on the newly constructed directed acyclic graph DAG(*d*), the semantic contribution of an ancestor node *d*_s_ to the disease *d* can be calculated as follows:

(2) $$\begin{array}{c} D_{d}\left(d_{\text{s}}\right)=\left\{\begin{array}{cc} 1, & \text{if}\;\;d_{\text{s}}=d,\\ \max\left\{\Delta *D_{d}\left(d^{\prime }\right)\;|\;d^{\prime }\in \text{children of }d_{\text{s}}\right\}, & \text{otherwise,} \end{array}\right.\\ \text{DV}\left(d\right)=\underset{d_{i}\in T\left(d\right)}{\sum }D_{d}\left(d_{i}\right), \end{array}$$

where Δ is the semantic contribution decay factor with value between 0 and 1. And, in addition, according to the experimental results of some previous state-of-the-art methods [57, 58], the most appropriate value for Δ will be 0.5. Hence, based on the assumption that two diseases with more common ancestor nodes in their *DAG*s shall have higher semantic similarity, the semantic similarity between two diseases *d*_*i*_ and *d*_*j*_ can be defined as follows:

(3) $$\begin{array}{c} \begin{array}{c} \text{DisSemSim}\left(i,j\right)=\frac{\sum_{t\in T\left(d_{i}\right)\cap T\left(d_{j}\right)}\left(D_{d_{i}}\left(t\right)+D_{d_{j}}\left(t\right)\right)}{\text{DV}\left(d_{i}\right)+\text{DV}\left(d_{j}\right)}. \end{array} \end{array}$$

#### 2.6.2. Calculation of the Gaussian Interaction Profile Kernel Similarity for Diseases (GIPSim)

Based on the hypothesis that functionally similar genes are often associated with similar diseases, in this section, we will adopt the Gaussian Interaction Profile Kernel to calculate the similarity of diseases according to the following steps:

Firstly, based on the networks *G*_1_ and *G*_2_ constructed above, for any given lncRNA *l*_*i*_ and disease *d*_*j*_, we define that

(4) $$\begin{array}{c} Y_{1}\left(i,j\right)=\left\{\begin{array}{cc} 1, & \text{if}\;\;\left(l_{i},d_{j}\right)\in G_{1},\\ 0, & \text{otherwise}. \end{array}\right. \end{array}$$

Next, for any given miRNA *m*_*i*_ and disease *d*_*j*_, we define that

(5) $$\begin{array}{c} Y_{2}\left(i,j\right)=\left\{\begin{array}{cc} 1, & \text{if}\;\;\left(m_{i},d_{j}\right)\in G_{2},\\ 0, & \text{otherwise}. \end{array}\right. \end{array}$$

Hence, let IP_*l*_(*d*_*i*_) denote the *i*th column of the matrix *Y*_1_, then we can calculate the Gaussian Kernel Similarity between diseases *d*_*i*_ and *d*_*j*_ based on their interaction profiles as follows:

(6) $$\begin{array}{c} \begin{array}{c} {\text{GIP}}_{\text{LDSIM}}\left(i,j\right)=\exp\left(-\gamma_{d1}{\left\|{\text{IP}}_{l}\left(d_{i}\right)-{\text{IP}}_{l}\left(d_{j}\right)\right\|}^{2}\right), \end{array} \end{array}$$

(7) $$\begin{array}{c} \gamma_{d1}=\frac{1}{\left(1/n_{d1}\right)\sum_{i=1}^{n_{d1}}\left\|\text{IP}{\left(d_{l}\left(i\right)\right)}^{2}\right\|}, \end{array}$$

where the parameter *n*_*d*1_ denotes the number of different diseases in *G*_1_.

In a similar way, let IP_*m*_(*d*_*i*_) denote the *i*th column of matrix *Y*_2_, then we can calculate the Gaussian Kernel Similarity between diseases *d*_*i*_ and *d*_*j*_ based on their interaction profiles as follows:

(8) $$\begin{array}{c} {\text{GIP}}_{\text{MDSIM}}\left(i,j\right)=\exp\left(-\gamma_{d2}{\left\|{\text{IP}}_{m}\left(d_{i}\right)-{\text{IP}}_{m}\left(d_{j}\right)\right\|}^{2}\right),\\ \gamma_{d2}=\frac{1}{\left(1/n_{d2}\right)\sum_{i=1}^{n_{d2}}{\left\|{\text{IP}}_{m}\left(d_{i}\right)\right\|}^{2}}, \end{array}$$

Here, the parameter *n*_*d*2_ denotes the number of different diseases in *G*_2_.

Thereafter, based on these above formulas, we can calculate the Gaussian Interaction Profile Kernel Similarity between diseases *d*_*i*_ and *d*_*j*_ as follows:

(9) $$\begin{array}{c} \text{GIPSim}\left(i,j\right)=\frac{{\text{GIP}}_{\text{LDSIM}}\left(i,j\right)+{\text{GIP}}_{\text{MDSIM}}\left(i,j\right)}{2}. \end{array}$$

### 2.7. Calculation of the Similarity of lncRNA Pairs (lncSim)

#### 2.7.1. Calculation of the lncRNA Functional Similarity (lncfunSim)

For any two given lncRNAs such as *l*_*i*_ and *l*_*j*_, let DT_1_={dt_11_, dt_12_,…, dt_1*m*_} be all the diseases related to *l*_*i*_ in *G*_1_ and DT_2_={dt_21_, dt_22_,…, dt_2*n*_} be all the diseases related to *l*_*j*_ in *G*_1_, then we can define the functional similarity between *l*_*i*_ and *l*_*j*_ as follows:

(10) $$\begin{array}{c} \text{lncfunSim}\left(i,j\right)=\frac{\sum_{1\leq k\leq m}\text{SemSims}\left({\text{dt}}_{1k},{\text{DT}}_{2}\right)+\sum_{1\leq k\leq n}\text{SemSims}\left({\text{dt}}_{2k},{\text{DT}}_{1}\right)}{m+n}, \end{array}$$

where

(11) $$\begin{array}{c} \text{SemSims}\left({\text{dt}}_{1k},{\text{DT}}_{2}\right)=\max_{1\leq l\leq n}\left(\text{DisSemSim}\left({\text{dt}}_{1k},{\text{dt}}_{2l}\right)\right),\\ \text{SemSims}\left({\text{dt}}_{2k},{\text{DT}}_{1}\right)=\max_{1\leq l\leq m}\left(\text{DisSemSim}\left({\text{dt}}_{2k},{\text{dt}}_{1l}\right)\right). \end{array}$$

#### 2.7.2. Calculation of the Gaussian Interaction Profile Kernel Similarity for lncRNAs (GIP_lncSim_)

For any two given lncRNAs such as *l*_*i*_ and *l*_*j*_, similar to the definition of formula (6), let IP(*l*_*i*_) and IP(*l*_*j*_) denote the *i*th and the *j*th row of the matrix *Y*_1_, respectively, then we can calculate the Gaussian Kernel Similarity between diseases *l*_*i*_ and *l*_*j*_ based on their interaction profiles as follows:

(12) $$\begin{array}{c} {\text{GIP}}_{\text{lncSim}}\left(i,j\right)=\exp\left(-\gamma_{l}{\left\|\text{IP}\left(l_{i}\right)-\text{IP}\left(l_{j}\right)\right\|}^{2}\right),\\ \gamma_{l}=\frac{1}{\left(1/n_{l}\right)\sum_{i=1}^{n_{l}}{\left\|\text{IP}\left(l_{i}\right)\right\|}^{2}}, \end{array}$$

where *n*_*l*1_ denotes the number of different lncRNAs in *G*_1_.

Hence, based on these formulas given above, we can finally define the similarity measurement between lncRNAs *l*_*i*_ and *l*_*j*_ as follows:

(13) $$\begin{array}{c} \text{lncSim}\left(i,j\right)=\frac{\text{lncfunSim}\left(i,j\right)+{\text{GIP}}_{\text{lncSim}}\left(i,j\right)}{2}. \end{array}$$

### 2.8. Calculation of the Similarity between miRNAs (miRSim)

#### 2.8.1. Calculation of the miRNA Function Similarity (miRfunSim)

For any two given miRNAs, such as *m*_*i*_ and *m*_*j*_, let DT_3_={dt_31_, dt_32_,…, dt_3*p*_} be all the diseases related to *m*_*i*_ in *G*_2_ and DT_4_={dt_41_, dt_42_,…, dt_4*q*_} be all the diseases related to *m*_*j*_ in *G*_2_, then we can define the functional similarity between *m*_*i*_ and *m*_*j*_ as follows:

(14) $$\begin{array}{c} \text{miRfunSim}\left(i,j\right)=\frac{\sum_{1\leq k\leq p}\text{SemSims}\left({\text{dt}}_{1k},{\text{DT}}_{3}\right)+\sum_{1\leq k\leq p}\text{SemSims}\left({\text{dt}}_{2k},{\text{DT}}_{4}\right)}{p+q}. \end{array}$$

#### 2.8.2. Calculation of the Gaussian Interaction Profile Kernel Similarity for miRNAs (GIP_miRSim_)

For any two given miRNAs, such as *m*_*i*_ and *m*_*j*_, in a similar way, let IP(*m*_*i*_) and IP(*m*_*j*_) represent the *i*th and *j*th row in matrix *Y*_2_, respectively, then we can calculate the Gaussian Kernel Similarity between diseases *m*_*i*_ and *m*_*j*_ based on their interaction profiles as follows:

(15) $$\begin{array}{c} {\text{GIP}}_{\text{miRSim}}\left(i,j\right)=\exp\left(-\gamma_{m}{\left\|\text{IP}\left(m_{i}\right)-\text{IP}\left(m_{j}\right)\right\|}^{2}\right),\\ \gamma_{m}=\frac{1}{\left(1/n_{m}\right)\sum_{i=1}^{n_{m}}{\left\|\text{IP}\left(m_{i}\right)\right\|}^{2}}, \end{array}$$

where *n*_*m*2_ denotes the number of miRNAs in *G*_2._

Hence, based on these formulas presented above, we can finally define the similarity measurement between miRNAs *m*_*i*_ and *m*_*j*_ as follows:

(16) $$\begin{array}{c} \text{miRSim}\left(i,j\right)=\frac{\text{miRfunSim}\left(i,j\right)+{\text{GIP}}_{\text{miRSim}}\left(i,j\right)}{2}. \end{array}$$

### 2.9. Weighted *K* Nearest Neighbor Profiles for Diseases, lncRNAs, and miRNAs (WKNNP)

Let *D*={*d*_1_, *d*_2_,…, *d*_*m*_}, *L*={*l*_1_, *l*_2_,…, *l*_*n*_}, and *M*={*m*_1_, *m*_2_,…, *m*_*k*_} denote the set of *m* diseases, *n* lncRNAs, and *k* miRNAs, respectively. Let *T*(*d*_*i*_)=*T*(*i*,:,:) denote the *i*th horizontal slice matrix in disease axis of the tensor *T*, hence, *T*(*d*_*i*_) also represents the interaction profile for the disease *d*_*i*_. Let *T*(*l*_*j*_)=*T*(:,*j*,:) denote the *j*th lateral slice matrix in lncRNA axis of the tensor *T*, hence, *T*(*l*_*j*_) also represents the interaction profile for lncRNA *l*_*j*_. Let *T*(*m*_*p*_)=*T*(:,:,*p*) denote the *p*th frontal slice matrix in miRNA axis of the tensor *T*, hence, *T*(*m*_*p*_) also denotes the interaction profile for miRNA *m*_*p*_. Then, it is obvious that the values in these three kinds of interaction profiles of any novel diseases, lncRNAs, or miRNAs are all zeros, which may lead to unsatisfactory prediction performance during inferring potential associations between diseases and lncRNA-miRNA pairs. Hence, in this section, we will perform a procedure for the construction of new interaction profiles to address the problem mentioned above. And, in this procedure, for each disease *d*_*i*_, its association with other *K* nearest known diseases (including at least one experimentally verified association) and corresponding *K* interaction profiles will be utilized to obtain the following interaction profile:

(17) $$\begin{array}{c} T_{D}\left(d_{i}\right)=\frac{1}{Q_{d}}\overset{K}{\underset{t=1}{\sum }}w_{t}T\left(d_{t},:,:\right), \end{array}$$

where, {*d*_1_, *d*_2_,…, *d*_*K*_} are the diseases sorted in descending order based on their similarity to *d*_*i*_, *w*_*t*_ is the weight coefficient, and *w*_*t*_=*α* ^*t*−1^ *∗*disSim(*d*_*t*_, *d*_*i*_), which means that a higher weight will be assigned if *d*_*t*_ is more similar to *d*_*i*_. The parameter α is a decay term with values between 0 and 1. The parameter *Q*_*d*_ is a normalization term, and there is *Q*_*d*_=∑_*t*=1_^*K*^disSim(*d*_*t*_, *d*_*i*_).

In the same manner, the new interaction profile for each *l*_*k*_ can be determined as follows:

(18) $$\begin{array}{c} T_{L}\left(l_{k}\right)=\frac{1}{Q_{l}}\overset{K}{\underset{t=1}{\sum }}w_{t}T\left(:,l_{t},:\right), \end{array}$$

where {*l*_1_, *l*_2_,…, *l*_*K*_} are the lncRNAs sorted in the descending order based on their similarity to *l*_*k*_, *w*_*t*_ is the weight coefficient, and *w*_*t*_=*α* ^*t*−1^ *∗*lncSim(*l*_*t*_, *l*_*k*_), which means that a higher weight will be assigned if *l*_*t*_ is more similar to *l*_*k*_. The parameter *Q*_*l*_ is a normalization term, and there is *Q*_*l*_=∑_*t*=1_^*K*^lncSim(*l*_*t*_, *l*_*k*_).

Similarly, the new interaction profile for each *m*_*p*_ can be determined as follows:

(19) $$\begin{array}{c} T_{M}\left(m_{p}\right)=\frac{1}{Q_{m}}\overset{K}{\underset{t=1}{\sum }}w_{t}T\left(:,:,m_{p}\right), \end{array}$$

where {*m*_1_, *m*_2_,…, *m*_*K*_} are the miRNAs sorted in the descending order based on their similarity to *m*_*p*_, *w*_*t*_ is the weight coefficient, and *w*_*t*_=*α* ^*t*−1^ *∗*miRSim(*m*_*t*_, *m*_*p*_), which means that a higher weight is assigned if *m*_*t*_ is more similar to *m*_*p*_. The parameter *Q*_*m*_ is a normalization term, and there is *Q*_*m*_=∑_*t*=1_^*K*^miRSim(*m*_*t*_, *m*_*p*_).

Thereafter, after combining the above three kinds of tensors *T*_*D*_, *T*_*L*_, and *T*_*M*_ obtained from different data spaces and replacing *T*(*i*, *j*, *k*)=0 with an associated likelihood score, we can update the original adjacency matrix *T* as follows:

(20) $$\begin{array}{c} T=\max\left(T,T_{DLM}\right), \end{array}$$

where *T*_*DLM*_=(*a*_1_ *T*_*D*_+*a*_2_ *T*_*L*_+*a*_3_ *T*_*M*_/∑*a*_*i*_), (*i*=1,2,3).

### 2.10. PADLMHOOI

Inspired by the successful application of tensor decomposition in the field of link prediction and the application of nonnegative matrix decomposition methods in inferring disease-miRNA associations, in this section, we proposed a novel model called PADLMHOOI to predict new associations between diseases and miRNA-lncRNA pairs. From above descriptions, it is easy to know that a tensor is a multidimensional array. Currently, the most commonly used tensor decomposition techniques include Tucker decomposition [59], HOSVD [60], and HOOI [61]. In this section, we will perform Tucker decomposition on above constructed tensor *T*. Assuming *T* ∈ *ℝ* ^*n*_1_×*n*_2_×*n*_3_^, the tucker decomposition aims at finding *Z*_*α*_(*α* ∈ (1,2,3)) and core tensor *G* ∈ *ℝ* ^*R*_1_×*R*_2_×*R*_3_^ that can solve the following optimization problem:

(21) $$\begin{array}{c} \begin{array}{cc} \text{minimize} & {\left\|T-\hat{T}\right\|}_{F}^{2},\\ \text{s}.\text{t}. & {\hat{T}}^{i,j,k}=\underset{r_{1},r_{2},r_{3}}{\sum }Z_{1}^{i,r_{1}}Z_{2}^{j,r_{2}}Z_{3}^{k,r_{3}}G^{r_{1},r_{2},r_{3}},\;\forall \;\;i,j,k. \end{array} \end{array}$$

Hence, based on formula (21), we can further transform this equation to following simple form:

(22) $$\begin{array}{c} \begin{array}{cc} \text{minimize} & {\left\|T-\hat{T}\right\|}_{F}^{2},\\ \text{s}.\text{t}. & \hat{T}=GX_{1}Z_{1}X_{2}Z_{2}X_{3}Z_{3}=⟦G;Z_{1},Z_{2},Z_{3}⟧, \end{array} \end{array}$$

*Z*_1_ ∈ *ℝ* ^*n*_1_×*R*_1_^, *Z*_2_ ∈ *ℝ* ^*n*_2_×*R*_2_^, and *Z*_3_ ∈ *ℝ* ^*n*_3_×*R*_3_^ are the factor matrices, which are usually orthogonal and can be considered as the main component of each mode. *R*_1_, *R*_2_, and *R*_3_ are the number of columns (max(*R*_1_, *R*_2_, *R*_3_) ≪ min(*n*_1_, *n*_2_, *n*_3_)) in the factor matrices *Z*_1_, *Z*_2_, and *Z*_3_ respectively. The notation *X*_*n*_ denotes *n*-mode product; ⟦*G*; *Z*_1_, *Z*_2_, *Z*_3_⟧ is the shorthand introduced by Kolda and Gibson [62] (Supplementary File A).

Based on equation (22), the above optimization problem can be solved according to the following steps:

Considering that the derivation forms of *Z*_1_, *Z*_2_, and *Z*_3_ are similar, we will only derive the iterative formula of *Z*_1_ as an example. Firstly, as illustrated in formula (23), the objective function given in formula (22) can be rewritten as a matrix form of *T* along the first dimension:

(23) $$\begin{array}{c} {\left\|T_{\left(1\right)}-Z_{1}{⟦G;Z_{2},Z_{3}⟧}_{\left(1\right)}\right\|}_{F}^{2}, \end{array}$$

where *T*_(1)_ ∈ *ℝ* ^*n*_1_×(*n*_2_*∗n*_3_)^ is the unfolding of *T* along the first dimension (Supplementary File A). Assuming that the optimal solution *Z*_1_ satisfies all the constraints in equation (22), we have

(24) $$\begin{array}{c} T_{\left(1\right)}=Z_{1}{⟦G;Z_{2},Z_{3}⟧}_{\left(1\right)}=Z_{1}G_{\left(1\right)}{\left(Z_{2}⊗Z_{3}\right)}^{T}, \end{array}$$

where ⊗ denotes the Kronecker product, and moreover, we have

(25) $$\begin{array}{c} S_{1}=G_{\left(1\right)}{\left(Z_{2}⊗Z_{3}\right)}^{T}. \end{array}$$

Hence, formula (24) can be regarded as a nonnegative matrix factorization (NMF) form [63]. Then, we can finally obtain the solution of *Z*_1_ by updating NMF as follows:

(26) $$\begin{array}{c} Z_{1}⟵Z_{1}*\frac{T_{\left(1\right)}S_{\left(1\right)}^{T}}{Z_{1}S_{\left(1\right)}S_{\left(1\right)}^{T}}. \end{array}$$

Hence, we can finally obtain the factor matrices *Z*_2_ and *Z*_3_ in a similar way. Thereafter, while fixing the factor matrices *Z*_1_, *Z*_2_, and *Z*_3_, the objective function in formula (22) can be converted to the following form:

(27) $$\begin{array}{c} {\left\|T-\hat{T}\right\|}_{F}^{2}={\left\|\text{vec}\left(T\right)-\left(Z_{3}⊗Z_{2}⊗Z_{1}\right)\text{vec}\left(G\right)\right\|}_{F}^{2}, \end{array}$$

where vec(·) denotes the vectorization of the tensor. And moreover, based on formula (27), the following linear equation can be obtained:

(28) $$\begin{array}{c} \text{vec}\left(X\right)=\left(Z_{3}⊗Z_{2}⊗Z_{1}\right)\text{vec}\left(G\right). \end{array}$$

Let *Q*=*Z*_3_ ⊗ *Z*_2_ ⊗ *Z*_1_, then obviously, formula (28) can also be regarded as a NMF, and thereafter, the core tensor in formula (28) can be obtained as follows [63]:

(29) $$\begin{array}{c} \text{vec}\left(G\right)⟵\text{vec}\left(G\right)*\frac{Q^{T}\text{vec}\left(T\right)}{Q^{T}Q\text{vec}\left(G\right)}=\text{vec}\left(G*\frac{⟦T;Z_{1}^{T},Z_{2}^{T},Z_{3}^{T}⟧}{⟦G;Z_{1}^{T},Z_{2}^{T},Z_{3}^{T}⟧}\right), \end{array}$$

(30) $$\begin{array}{c} G⟵G*\frac{⟦T;Z_{1}^{T},Z_{2}^{T},Z_{3}^{T}⟧}{⟦G;Z_{1}^{T},Z_{2}^{T},Z_{3}^{T}⟧}. \end{array}$$

Based on above formulas, the pseudocode of our prediction model PADLMHOOI based on tensor decomposition can be described as follows:

 
*Step 1*. **Input**: *T*, *R*_1_, *R*_2_, *R*_3_, *Z*_1_, *Z*_2_, *Z*_3_, *G*, and the convergence threshold *ε*.
 
*Step 2*. **Repeat**
      For *i* = 1 to 3:
       Update *Z*_*i*_ according to formula (26)
      End For
      Update *G* according to formula (30)
     **Until**‖*T* − [[*G*; *Z*_1_, *Z*_2_, *Z*_3_]]‖_*F*_^2^ < *ε*
 
*Step 3*. **Return** *Z*_1_, *Z*_2_, *Z*_3_, *G*

According to above steps, we can obtain the final predicted disease-lncRNA-miRNA association tensor *T* ^*∗*^=*GX*_1_ *Z*_1_ *X*_2_ *Z*_2_ *X*_3_ *Z*_3_, and after prioritizing the disease-related lncRNA-miRNA pairs based on the entities in the tensor *T* ^*∗*^, obviously, the top-ranked lncRNA-miRNA pairs can be regarded as more likely to be related to the corresponding disease.

## 3. Results and Analysis

### 3.1. Leave-One-Out Cross-Validation (LOOCV)

In order to estimate the prediction performance of our newly proposed prediction model, the global leave-one-out cross-validation (LOOCV), 2-fold cross-validation (2-fold CV), and 10-fold cross-validation (10-fold CV) were implemented on PADLMHOOI, respectively. In the *K*-fold cross-validation, the initial sample will be divided into *K* subsample sets, and a single subsample set is retained as the data for the validation model, while the other *K* − 1 samples are used to train the model. During simulation, the cross-validation will be performed *K* times, and each subsampling set will be verified once, and the average results of *K* times will be utilized to obtain a single estimation. Moreover, in order to reduce the performance deviation caused by the random sample partitioning, we divide the partition 100 times and then obtain the ROC curve and the AUC value in the same way as the LOOCV. And, as a result, from the following Table 1, it is easy to see that PADLMHOOI can achieve reliable AUCs of 0.9545, 0.9730 ± 0.0119, and 0.9626 ± 0.0150 in the frameworks of global LOOCV, 2-fold CV, and 10-fold CV, respectively. Additionally, in order to further estimate the prediction performance of PADLMHOOI, we implemented it under the framework of local LOOCV, and the simulation results of 50 predicted related diseases were illustrated in Supplementary Table 4.

### 3.2. Performance Comparison with Other Methods

**Figure 2:**
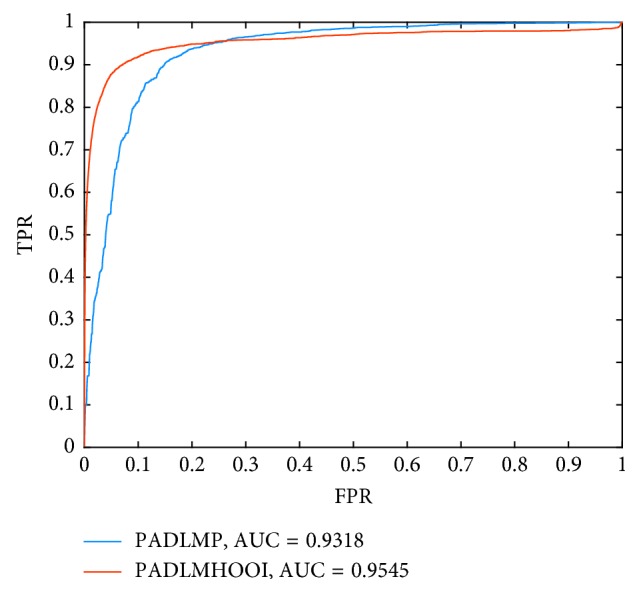
Performance comparison between PADLMHOOI and PADLMP in terms of ROC curves and AUCs based on the 3047 known disease-lncRNA-miRNA associations.

To the best of our knowledge, up to now, PADLMP [39] is the unique model having been proposed for predicting potential associations between disease and lncRNA-miRNA pairs, in which, these three kinds of nodes such as disease nodes, lncRNA nodes, and miRNA nodes are considered simultaneously to construct a triple network. And, the major difference between PADLMP and our model PADLMHOOI is that PADLMP is based on the method of link prediction. Therefore, in order to compare PADLMP with our model PADLMHOOI, we implemented LOOCV to verify the prediction performance of these two models based on the 3047 known disease-lncRNA-miRNA associations downloaded above. In the first experiment, we set the parameters in PADLMP to their best values; specifically, the step size *K* is set to 2 and the attenuation coefficient *γ* is set to 0.01. Meanwhile, for convenience, we set the parameters in PADLMHOOI as follows: the parameters *a*_1_, *a*_2_, and *a*_3_ in formula (20) are all set to 1, the parameters *r*_1_, *r*_2_, and *r*_3_ in formula (21) are all set to 5, and the parameters *K* and *α* in formulas (17)–(19) are all set to 3 and 0.1 separately. And, as illustrated in Figure 2, it is easy to see that PADLMHOOI and PADLMP can achieve the AUCs of 0.9545 and 0.9318 separately, which demonstrate that the prediction performance of PADLMHOOI is superior to that of PADLMP.

**Figure 3:**
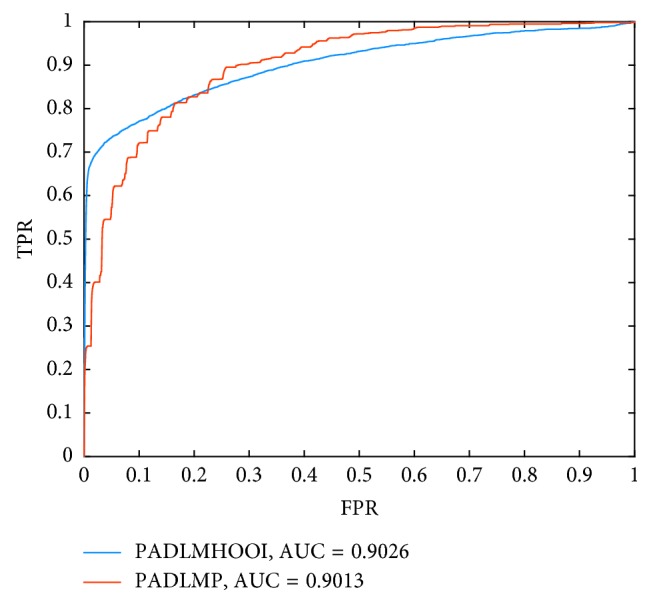
Performance comparison between PADLMHOOI and PADLMP in terms of ROC curves and AUCs based on the latest 3678 known disease-lncRNA-miRNA associations. Here, comparing with the AUCs in Figure 2, the reason that the AUCs of our model decline in Figure 3 is that the values of parameters *K* and *α* are different. In Figure 2, *K* = 3 and *α* = 0.1, while in Figure 3, *K* = 10 and *α* = 0.5.

As time went by, we found that some databases have been updated. Hence, in order to further demonstrate the advancement of PADLMHOOI, we once again collected the latest disease-lncRNA correlations from the databases lnc2cancer v2.0, lncRNADisease 2.0 [64], and MNDR v2.0 [48], collected the latest disease-miRNA associations from the database HMDD v3.0, and collected the latest lncRNA-miRNA associations from the database RAID v2.0 [65] separately. And thereafter, we reconstructed the triple network based on these newly collected latest datasets. In the newly constructed triple network, the numbers of disease nodes, lncRNA nodes, and miRNA nodes are 42, 234, and 251 respectively; the number of known associations between diseases and lncRNA-miRNA pairs is 3,768; the number of known associations between diseases and lncRNAs is 733; and the number of known associations between diseases and miRNAs is 674. Then, based on the new triple network, we compared our model PADLMHOOI with PADLMP once more. And, in this second experiment, we set the parameters *K* and *α* to 10 and 0.5, respectively, in PADLMHOOI and kept other parameters unchanged as in the first experiment. And, as illustrated in Figure 3, simulation results show that PADLMHOOI and PADLMP can achieve AUCs of 0.9026 and 0.9013, respectively, which demonstrate that the prediction performance of PADLMHOOI outperforms that of PADLMP markedly.

**Figure 4:**
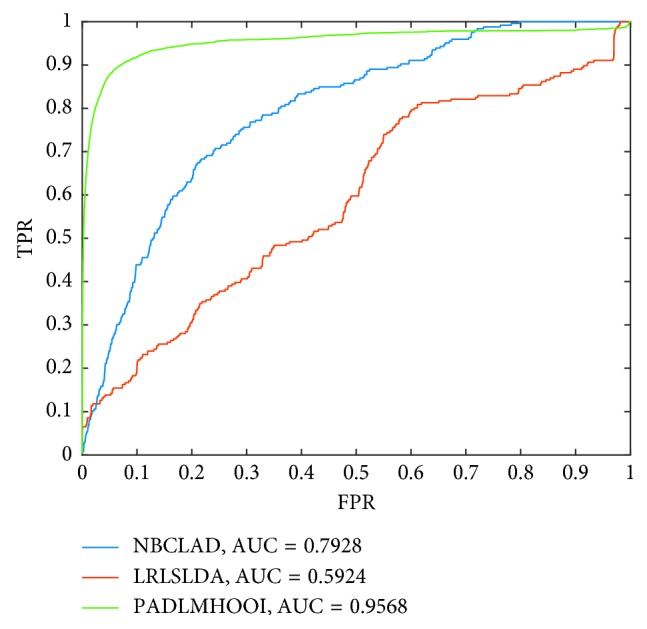
The comparison results between PADLMHOOI and LRLSLDA and NBCLAD.

**Figure 5:**
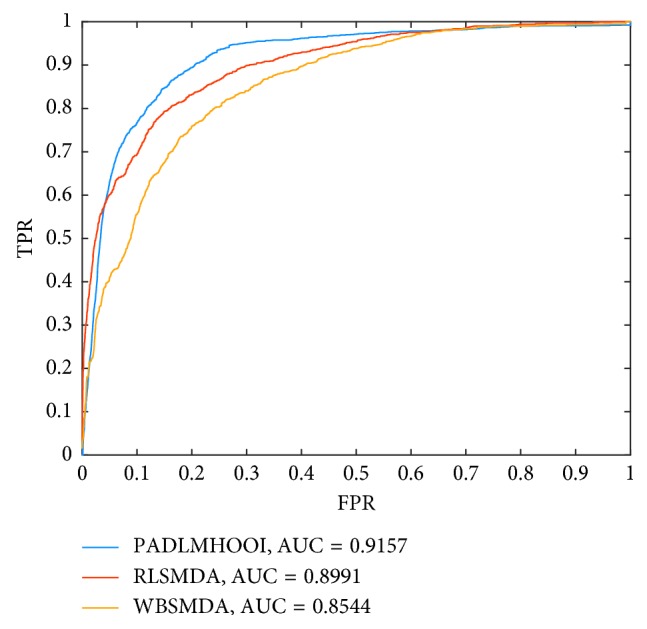
The comparison results between PADLMHOOI and RLSMDA and WBMDA.

Additionally, the interesting point is that our model can infer potential disease-lncRNA associations and disease-miRNA associations incidentally, while predicting potential associations between diseases and lncRNA-miRNA pairs. Hence, it is reasonable as well to compare our model PADLMHOOI with prediction models for inferring potential disease-lnRNA or disease-miRNA associations. Therefore, in this section, we would compare PADLMHOOI with some state-of-the-art computational prediction models such as the LRLSLDA [26], NBCLAD [25], WBSMDA [66], and RLSMDA [67]. Among them, LRLSLDA is a semisupervised learning-based prediction model for inferring potential lncRNA-disease associations; NBCLAD is a probabilistic model for predicting potential associations between diseases and lncRNAs; WBSMDA is a prediction model for predicting potential associations between diseases and miRNAs; and RLSMDA is a prediction model for predicting disease-related miRNAs based on the framework of regularized least squares. In addition, while comparing with LRSLDA, known disease-lncRNA associations were obtained from the triple disease-lncRNA-miRNA network; however, the parameters in LRSLDA are set to the same values given in the literature. Moreover, while comparing with NBCLDA, considering that there are four kinds of nodes such as diseases, lncRNAs, miRNAs, and genes included in NBCLDA, there are three kinds of nodes such as diseases, lncRNAs, and miRNAs in our model PADLMHOOI. Hence, for the sake of fairness, we only compared PADLMHOOI with the submethod NBCLDA-GN1-SD. And, as illustrated in Figure 4, simulation results show that PADLMHOOI, NBCLDA-G1-SD, and LRSLDA can achieve AUCs of 0.9568, 0.7928, and 0.5924 separately, which demonstrate that PADLMHOOI thoroughly defeats both NBCLDA-G1-SD and LRSLDA. In addition, while comparing with WBSMDA and RLSMDA, 674 known disease-miRNA associations were obtained from the triple disease-lncRNA-miRNA network; however, the parameters in both WBSMDA and RLSMDA are set to the same values given in the literatures. And, as illustrated in Figure 5, simulation results show that PADLMHOOI, WBSMDA, and RLSMDA can achieve AUCs of 0.9157, 0.8544, and 0.8991, respectively, which demonstrate that PADLMHOOI outperforms both WBSMDA and RLSMDA thoroughly as well.

### 3.3. Recall Ratio Analysis

**Figure 6:**
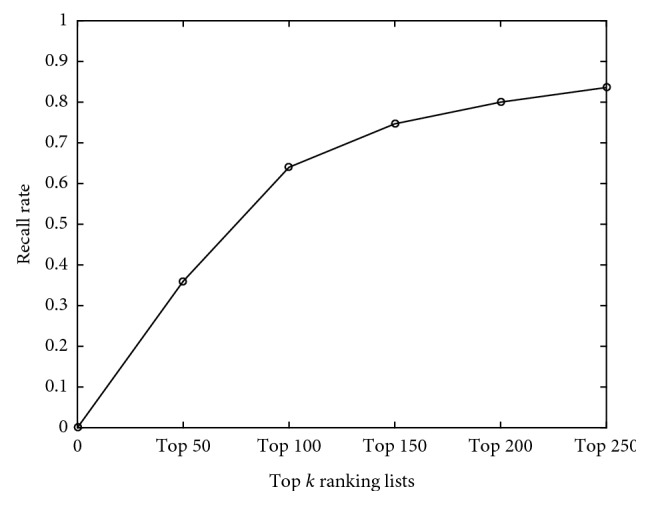
The recall rate of all the selected diseases in different top *k* ranking lists.

In this section, in order to further evaluate the prediction performance of PADLMHOOI, we compared the recall value of PADLMHOOI and other state-of-the-art models. It is well known that the higher recall ratio of all selected diseases in a top *k* ranking list means that the more positive testing samples (real disease-related lncRNA-miRNA pairs) have been identified successfully. And, as a result, Figure 6 illustrates the recall rate of all selected diseases in different top *k* ranking lists. Moreover, we further listed the recall rate of some given diseases associated with at least 80 verified lncRNA-miRNA associations in Supplementary Table 5.

### 3.4. Case Studies

In this section, case studies of breast neoplasms, colon neoplasms, and prostate neoplasms were conducted to further verify the capability of PADLMHOOI to detect novel associations between diseases and lncRNA-miRNA pairs separately. And, among these three kinds of case studies, breast cancer is the second leading cause of female cancer death and comprises 22% of all cancers in women [68, 69]. The related literature has suggested that lncRNAs and miRNAs play an important role in the formation of many diseases, and the formation of breast cancer may be more relevant to them [70, 71]. Predicting breast cancer-associated lncRNA-miRNA pairs and identifying lncRNAs and miRNAs as biomarkers may make a significant contribution to better diagnosis and treatment of breast cancer [71]. In Supplementary Table 6, we have listed the top 30 candidate lncRNA-miRNA pairs related to breast cancer. And, in Supplementary Table 6, the column of lncRID and miRID denotes lncRNA ID and miRNA ID, respectively. Evi1 and Evi2 denote some authority database or published literature containing verified disease-lncRNA or disease-miRNA associations separately. “#” and “∗” stand for databases of lncRNADisease and MNDR v2.0, respectively, which consist of known disease-lncRNA associations or contain published literatures to support the association between predicted lncRNAs and breast cancer. “!,” “&,” and “+” stand for databases of HMDD, miR2Disease, and miRCancer, respectively, which consist of known disease-miRNA associations or contain published literature to support the association between predicted miRNAs and breast cancer. Particularly, “Nan” indicates that there is no database or no published literature to support the predicted results. From Supplementary Table 6, it is easy to see that all candidate disease-lncRNA associations have been verified in databases of the lncRNADisease and MNDR v2.0 or published papers containing these databases. And, in addition, there are 42 out of 50 candidate disease-miRNA associations having been reported by HMDD, miR2Disease, and miRCancer or published paper containing these databases. Moreover, we discovered that those novel miRNAs with miRID 35, 51, 73, 164, and 186 are related to some important factors affecting the development of breast neoplasms. Hence, it is obvious that we infer that these lncRNA-miRNA pairs may be associated with breast cancer.

In addition, colonic tumors are a type of malignancy that is common in the rectum and sigmoid borders [72]. Early colon cancer is difficult to detect because of its insignificant symptoms [73]. Unfortunately, the related literature reports that its incidence has been on the rise in recent years [74]. Therefore, predicting potential miRNAs and lncRNAs associated with colon tumors is of great significance for the diagnosis of early colon cancer. In Supplementary Table 7, we have listed the top 30 candidate lncRNA-miRNA pairs predicted to be associated with colon tumors. Moreover, all of these candidate lncRNAs and most of these candidate miRNAs have been verified by lncRNADisease database and MNDR v2.0, respectively.

Moreover, prostate neoplasm is one of the most common cancers in white and African-American men, and it is reported that there are about one in six white men and one in five African-American men having prostate cancer in their lifetime. Recent researches have shown that prostate neoplasm is caused by the malignancy of prostate epithelial cells [75], its formation includes many factors such as age, family history, and race [76], and particularly, some miRNAs such as has-let-7a-5p and lncRNAs such as XIST have been found to be involved in the formation of prostate neoplasms successively. Hence, it is interesting to infer potential miRNAs and lncRNAs associated with prostate neoplasms. In Supplementary Table 8, we have listed the top 30 prostate neoplasm-related candidate lncRNA-miRNA pairs. Moreover, all of these candidate lncRNAs and most of these candidate miRNAs have been verified by lncRNADisease and MNDR v2.0, respectively.

### 3.5. Parameter Sensitivity Analysis

Considering that there are some key parameters such as *K* and *α*, which may be significant to the performance of our prediction model PADLMHOOI, in this section, we will further estimate the effects of these key parameters to the prediction performance of PADLMHOOI. Firstly, we varied *K* from 1 to 10 during simulation. And, as a result, Table 2 illustrates the impacts of parameter *K* on the performance of PADLMHOOI. By observing Table 2, it is obvious that PADLMHOOI can achieve the maximum AUC value of 0.9708 while *K* = 8. And additionally, as for the impacts of the parameter *α*, considering the time costs, we set *K* = 3 and varied *α* from 0.1 to 0.9 during simulation. And as a result, Table 3 illustrates the impacts of parameter *α* on the performance of PADLMHOOI. By observing Table 3, it is obvious that PADLMHOOI can achieve the maximum AUC value of 0.9591 while *α* = 0.7.

## 4. Discussion and Conclusion

Researches on prediction of potential associations between lncRNA-miRNA pairs and diseases not only are helpful in understanding the disease mechanisms on lncRNA and miRNA levels but also play an important role in the detection of disease biomarkers, diagnosis, prognosis, and prevention. However, to our knowledge, although there are many researches having demonstrated that lncRNA-miRNA interactions are associated with the development of complex diseases, up to now, there are few models having been proposed for large-scale forecasting potential associations between diseases and lncRNA-miRNA pairs. Since traditional biological experiments are quite expensive and time-consuming, in this paper, based on the existing disease-miRNA associations, disease-lncRNA associations, lncRNA-miRNA interactions, and the assumption that genes with similar functions are often associated with similar diseases; we firstly constructed a three-order tensor *T* by adopting the method of WKNNP, and then, based on the method of tensor factorization, we further proposed a prediction model called PADLMHOOI to infer potential relations between diseases and lncRNA-miRNA pairs. And thereafter, simulation results under the frameworks of global and local LOOCV, 2-fold CV, and 10-fold CV, all confirmed the superiority of PADLMHOOI. Moreover, case studies of breast neoplasms, colon neoplasms, and prostate neoplasms further demonstrate that our model PADLMHOOI is an effective method for predicting potential disease-associated lncRNA-miRNA pairs. Certainly, there are still some limitations in PADLMHOOI. For example, although a large number of datasets have been integrated in PADLMHOOI, the amount of data available is still not enough; it is obvious that the prediction performance of PADLMHOOI will be better if more datasets can be collected. And in addition, in this paper, we only predicted the association between disease and a single lncRNA-miRNA pair. In the future, we will further modify PADLMHOOI to predict potential associations between diseases and multiple lncRNA-miRNA pairs.

## Figures and Tables

**Table 1:** Performance of PADLMHOOI in global LOOCV, 2-fold cross-validation, and 10-fold cross-validation.

Global LOOCV	2-fold CV	10-fold CV
0.9545	0.9730 ± 0.0119	0.9626 ± 0.0150

**Table 2:** Impacts of the parameter *K* on the performance of PADLMHOOI.

*K*	1	2	3	4	5	6	7	8	9	10
AUC	0.9660	0.9649	0.9591	0.9607	0.9666	0.9657	0.9675	0.9708	0.9703	0.9703

**Table 3:** Impacts of the parameter *α* on the performance of PADLMHOOI.

*α*	0.1	0.2	0.3	0.4	0.5	0.6	0.7	0.8	0.9
AUC	0.9545	00.9565	0.9582	0.9586	0.9583	0.9585	0.9591	0.9587	0.9539

## Data Availability

The data used to support the findings of this study are available from the corresponding author upon request.

## Abbreviations

PADLMHOOI: Prediction of potential associations between diseases and lncRNA-miRNA pairs based on the higher-order orthogonal iteration.

## Supplementary Materials

Supplementary 1 File 1: introduction to tensor and optimization of objective function and update of factor matrix and core tensor.

Supplementary 2 Table 1: 2048 known disease-lncRNA associations.

Supplementary 3 Table 2: 4041 known disease-miRNA associations.

Supplementary 4 Table 3: 20324 known lncRNA-miRNA associations.

Supplementary 5 Table 4: AUC score for 50 diseases in the framework of local LOOCV.

Supplementary 6 Table 5: recall ratio of some important diseases.

Supplementary 7 Table 6: the candidate lncRNA-miRNA pairs associated with breast cancer. In addition, the LncRNADisease and MNDR v2.0 databases have confirmed that these lncRNAs or miRNAs are associated with breast cancer.

Supplementary 8 Table 7: the candidate lncRNA-miRNA pairs associated with colon cancer. In addition, the LncRNADisease and MNDR v2.0 databases have confirmed that these lncRNAs or miRNAs are associated with colon cancer.

Supplementary 9 Table 8: the candidate lncRNA-miRNA pairs associated with pprostate cancer. In addition, the LncRNADisease and MNDR v2.0 databases have confirmed that these lncRNAs or miRNAs are associated with colon cancer.
