# Supplementary material for: A Novel Method for Predicting Disease-Associated LncRNA-MiRNA Pairs Based on the Higher-Order Orthogonal Iteration
Source: Comput Math Methods Med. 2019 May 2;2019:7614850. doi: 10.1155/2019/7614850 (PMC6525924; doi:10.1155/2019/7614850)
Supplement: Supplementary 1 — File 1: introduction to tensor and optimization of objective function and update of factor matrix and core tensor. [file 7614850.f1.docx]

**Matricization: Transforming a Tensor into a Matrix.**

Matricization, also known as unfolding or flattening, is the process of reordering the elements of an N-way array into a matrix. For instance, a 2×3×4 tensor can be arranged as a 6×4 matrix or a 3 ×8 matrix, and so on. In this review, we consider only the special case of mode-n matricization because it is the only form relevant to our discussion. A more general treatment of matricization can be found in Kolda [1]. The mode-n matricization of a tensor $X\in R^{I_{1}\times I_{2}\times\ldots\times I_{N}}$ is denoted by $X_{(n)}$ and arranges the mode-n fibers to be the columns of the resulting matrix. Though conceptually simple, the formal notation is clunky. Tensor element $(i_{1},i_{2},...,i_{N})$ maps to matrix element $(i_{n}, j)$, where:

$$\begin{aligned} j=1+\sum_{k=1,k\neq n}^{N} \left( i_{k}-1 \right)J_{k}with J_{k}=\prod_{m=1,m\neq n}^{k-1} I_{m} \end{aligned}(1)$$

The concept is easier to understand using an example. Let the frontal slices of $X\in\mathbb{R}^{3\times4\times2}$ be

$$X_{1}=\left[ \begin{matrix} 1 \\ 2 \\ 3 \end{matrix} \begin{matrix} 4 \\ 5 \\ 6 \end{matrix} \begin{matrix} 7 \\ 8 \\ 9 \end{matrix} \begin{matrix} 10 \\ 11 \\ 12 \end{matrix} \right] X_{2}=\left[ \begin{matrix} 13 \\ 14 \\ 15 \end{matrix} \begin{matrix} 16 \\ 17 \\ 18 \end{matrix} \begin{matrix} 19 \\ 20 \\ 21 \end{matrix} \begin{matrix} 22 \\ 23 \\ 24 \end{matrix} \right]$$

Then the three mode-*n* unfoldings are

$$X_{\left( 1 \right)}=\left[ \begin{matrix} 1 \\ 2 \\ 3 \end{matrix} \begin{matrix} 4 \\ 5 \\ 6 \end{matrix} \begin{matrix} 7 \\ 8 \\ 9 \end{matrix} \begin{matrix} 10 \\ 11 \\ 12 \end{matrix} \begin{matrix} 13 \\ 14 \\ 15 \end{matrix} \begin{matrix} 16 \\ 17 \\ 18 \end{matrix} \begin{matrix} 19 \\ 20 \\ 21 \end{matrix} \begin{matrix} 22 \\ 23 \\ 24 \end{matrix} \right]$$

$$X_{\left( 2 \right)}=\left[ \begin{aligned} 1 \\ 4 \\ 7 \\ 10 \end{aligned} \begin{matrix} \begin{matrix} 2 \\ 5 \end{matrix} \\ \begin{matrix} 8 \\ 11 \end{matrix} \end{matrix} \begin{matrix} \begin{matrix} 3 \\ 6 \end{matrix} \\ \begin{matrix} 9 \\ 12 \end{matrix} \end{matrix} \begin{matrix} \begin{matrix} 13 \\ 16 \end{matrix} \\ \begin{matrix} 19 \\ 22 \end{matrix} \end{matrix} \begin{matrix} \begin{matrix} 14 \\ 17 \end{matrix} \\ \begin{matrix} 20 \\ 23 \end{matrix} \end{matrix} \begin{matrix} \begin{matrix} 15 \\ 18 \end{matrix} \\ \begin{matrix} 21 \\ 24 \end{matrix} \end{matrix} \right]$$

$$X_{(3)}= \left[ \begin{matrix} 1 & 2 & \begin{matrix} \ldots& 12 \end{matrix} \\ 13 & 14 & \begin{matrix} \ldots& 24 \end{matrix} \end{matrix} \right]$$

Last, we note that it is also possible to vectorize a tensor. Once again the ordering

of the elements is not important so long as it is consistent. In the example above, the

vectorized version is

$$\mathrm{vex}\left( X \right)=\left[ \begin{matrix} 1 \\ \begin{matrix} 2 \\ \vdots\end{matrix} \\ 24 \end{matrix} \right]$$

**Tensor Multiplication: The n-Mode Product***.*

The *n*-Mode Product, i.e., multiplying a tensor by a matrix (or a vector) in mode n. The n-mode (matrix) product of a tensor $X\in\mathbb{R}^{I_{1}\times I_{2}\times\ldots\times I_{N}}$ with a matrix $U\in\mathbb{R}^{J\times I_{n}}$ is denoted by $X\times_{n}U$ and is of size$I_{1}\times\ldots\times I_{n-1}\times J\times I_{n+1}\times\ldots\times I_{N}$. Elementwise, we have

$$\begin{aligned} \left( X\times_{n}U \right)_{i_{1}\ldots i_{n-1}ji_{n+1}\ldots i_{N}}=\sum_{i_{n}=1}^{I_{n}} x_{i_{1}i_{2}\ldots i_{N}}u_{ji_{n}} \end{aligned}\left（ 2 \right）$$

**Matrix Kronecker,Khatri-Rap, and Hadamard Products.**

In the next section we used the product of some matrices, here we give a brief description.

***Kronecker Product***

The Kronecker product of matrices ***A***∈$\mathbb{R}^{I\times J}$ and ***B***∈$\mathbb{R}^{K\times L}$ is denoted by ***A****⨂****B****.* The result is a matrix of size(*IK*)×(*JL*) and defined by

$$\begin{aligned} \mathbf{A}\bigotimes\mathbf{B}=\left( \begin{aligned} a_{11}\boldsymbol{B} \\ a_{21}\boldsymbol{B} \\ \vdots\\ a_{I1}\boldsymbol{B} \end{aligned} \begin{aligned} a_{12}\boldsymbol{B} \\ a_{22}\boldsymbol{B} \\ \vdots\\ a_{I2}\boldsymbol{B} \end{aligned} \begin{aligned} \cdots\\ \cdots\\ \ddots\\ \cdots\end{aligned} \begin{aligned} a_{1J}\boldsymbol{B} \\ a_{2J}\boldsymbol{B} \\ \vdots\\ a_{IJ}\boldsymbol{B} \end{aligned} \right)=\left( \boldsymbol{a}_{\boldsymbol{1}}\bigotimes\boldsymbol{b}_{\boldsymbol{1}} \boldsymbol{a}_{\boldsymbol{1}}\bigotimes\boldsymbol{b}_{\boldsymbol{2}} \boldsymbol{a}_{\boldsymbol{1}}\bigotimes\boldsymbol{b}_{\boldsymbol{3}}\boldsymbol{\cdots}\boldsymbol{a}_{\boldsymbol{J}}\bigotimes\boldsymbol{b}_{\boldsymbol{L-1}} \boldsymbol{a}_{\boldsymbol{J}}\bigotimes\boldsymbol{b}_{\boldsymbol{L}} \right) \end{aligned}\mathbf{(3)}$$

As an example of the utility of the Kronecker product, consider the following.

Let $X\in R^{I1\times I2\times\cdot\cdot\cdot\times IN}$ and $A^{(n)}\in R^{Jn\times In}$ for all $n \in\{1, . . .,N\}$. Then, for any $n \in\{1, . . .,N\}$, we have The Khatri-Rao product

$$\begin{aligned} Y = X\times_{1}A^{\left( 1 \right)}\times_{2}A^{\left( 2 \right)}\ldots\times_{N}A^{\left( N \right)} \Leftrightarrow Y\left( n \right)= A^{\left( n \right)}X_{\left( n \right)}\left( A^{\left( n \right)}\otimes\ldots\otimes A^{\left( n+1 \right)}\otimes A^{\left( n-1 \right)}\otimes\ldots\otimes A^{\left( 1 \right)} \right)^{T}\left( 4 \right) \end{aligned}$$

***Hadamard product***

Suppose matrices $A\in\mathbb{R}^{I\times J} and B\in\mathbb{R}^{I\times J}$, then the Hadamard product is denoted by

$$\begin{aligned} A*B=\left[ \begin{matrix} \begin{matrix} a_{11}b_{11} & a_{12}b_{12} \\ a_{21}b_{21} & a_{22}b_{22} \end{matrix} & \begin{matrix} \cdots& a_{1J}b_{1J} \\ \cdots& a_{2J}b_{2J} \end{matrix} \\ \begin{matrix} \vdots& \vdots\\ a_{I1}b_{I1} & a_{I2}b_{I2} \end{matrix} & \begin{matrix} \ddots& \vdots\\ \ldots& a_{IJ}b_{IJ} \end{matrix} \end{matrix} \right] \end{aligned}(5)$$

**TUCKER3 Factorization**

We assume$X\in\mathbb{R}^{I\times J\times K}$, The TUCKER3 factorization aims to find $Z_{\alpha},\left( \alpha\in\left( 1,2,3 \right) \right)and core tensor G$that solves the following optimization problem:

$$\begin{aligned} \mathrm{minimize}\left\| X-\hat{X} \right\|_{F}^{2} s.t.\hat{X}^{i,j,k}=\sum_{r_{1},r_{2},r_{3}} Z_{1}^{i,r_{1}}Z_{2}^{j,r_{2}}Z_{3}^{k,r_{3}}G^{r_{1},r_{2},r_{3}} \forall i,j,k \end{aligned}(6)$$

Then we transform this equation into tensor and matrix form:

$$\begin{aligned} \mathrm{minimize}\left\| X-\hat{X} \right\|_{F}^{2} s.t.\hat{X}= G\times_{1}Z_{1}\times_{2}Z_{2}\times_{3}Z_{3}=\left[ \left[ G;Z_{1},Z_{2},Z_{3} \right] \right] \end{aligned}(7)$$

Here, $Z_{1}\in\mathbb{R}^{I\times R_{1}},Z_{2}\in\mathbb{R}^{J\times R_{2}},Z_{3}\in\mathbb{R}^{K\times R_{3}}$ are the factor matrices (which are usually orthogonal) and can be thought of as the principal components in each mode, $R_{1},R_{2},R_{3}$are the number of components (i.e., columns,$\max\left( R_{1},R_{2},R_{3} \right)\ll min(I,J,K)$) in the factor matrices $Z_{1},Z_{2},Z_{3}$*.* The tensor $G\in\mathbb{R}^{R_{1}\times R_{2}\times R_{3}}$ is called the core tensor and its entries show the level of interaction between the different components. The last equality uses the shorthand $[[G;Z_{1},Z_{2},Z_{3}]]$ introduced in Kolda[1].

Next, we give an iterative derivation formula for the feature matrices $Z_{1}, Z_{2}, Z_{3}$ and core tensor G.

**Update of the feature matrix:**

Considering that the derivation forms of $Z_{1}, Z_{2}, and Z_{3}$are similar, here we only derive the iterative formula of$Z_{1}$.

The objective function in equation (5) can be rewritten as a matrixed form of X along the first dimension.

$$\begin{aligned} \left\| X_{\left( 1 \right)}-Z_{1}\left⟦ G;Z_{2},Z_{3} \right⟧_{\left( 1 \right)} \right\|_{F}^{2} \end{aligned}(8)$$

where，$X_{\left( 1 \right)}\in\mathbb{R}^{J\times K}$.

Assuming that the optimal solution $Z_{1}$ satisfies all the constraints in equation (7), then$\begin{aligned} X_{\left( 1 \right)}=Z_{1}\left⟦ G;Z_{2},Z_{3} \right⟧_{\left( 1 \right)}=Z_{1}S_{1} \end{aligned}(9)$

where

$$\begin{aligned} S_{1}=G_{\left( 1 \right)}\left( Z_{2}\otimes Z_{3} \right)^{T} \end{aligned}(10)$$

Therefore, Equation 9 can be regarded as a non-negative matrix factorization (NMF) form in the literature [2-3]. Then, use the NMF update method to solve $Z_{1}$:

$$\begin{aligned} Z_{1}\leftarrow Z_{1}*\frac{X_{\left( 1 \right)}S_{\left( 1 \right)}^{T}}{Z_{1}{S_{\left( 1 \right)}S}_{\left( 1 \right)}^{T}} \end{aligned}(11)$$

**Core tensor update:**

We fixed the feature matrix $Z_{1}，Z_{2}，Z_{3}$, and the objective function in equation (5) can be converted to:

$$\begin{aligned} \left\| X-\hat{X} \right\|_{F}^{2}=\left\| vec\left( X \right)-\left( Z_{3}\otimes Z_{2}\otimes Z_{1} \right)vec\left( G \right) \right\|_{F}^{2} \end{aligned}(12)$$

The following linear equation can be obtained from equation (12):$\begin{aligned} \mathrm{vec}\left( X \right)=\left( Z_{3}\otimes Z_{2}\otimes Z_{1} \right)vec\left( G \right) \end{aligned}(13)$

Suppose $Q=Z_{3}\otimes Z_{2}\otimes Z_{1}$ Equation (13) can also be transformed into an NMF model to update the core tensor according to the method in [3] :$\begin{aligned} \mathrm{vec}\left( G \right)\leftarrow vec\left( G \right)*\frac{Q^{T}vec\left( X \right)}{Q^{T}Qvec\left( G \right)}=vec\left( G \right)*\frac{vec\left( \left⟦ X;Z_{1}^{T},Z_{2}^{T},Z_{3}^{T} \right⟧ \right)}{vec\left( vec\left( \left⟦ G;Z_{1}^{T},Z_{2}^{T},Z_{3}^{T} \right⟧ \right) \right)}=vec\left( G*\frac{\left⟦ X;Z_{1}^{T},Z_{2}^{T},Z_{3}^{T} \right⟧}{\left⟦ G;Z_{1}^{T},Z_{2}^{T},Z_{3}^{T} \right⟧} \right) \end{aligned}(14)$

By formula(14) $\begin{aligned} G\leftarrow G*\frac{\left⟦ X;Z_{1}^{T},Z_{2}^{T},Z_{3}^{T} \right⟧}{\left⟦ G;Z_{1}^{T},Z_{2}^{T},Z_{3}^{T} \right⟧} \end{aligned}(15)$

Reference

[1] T. G. Kolda, *Multilinear Operators for Higher-Order Decompositions*, Tech. Report SAND2006-2081, Sandia National Laboratories, Albuquerque, NM, Livermore, CA, 2006.

[2] D. D. Lee, H. S. Seung, Learning the parts of objects by non-negative matrix factorization. Nature 401, 788 (1999).

[3] Lee D D，Seung H S． Algorithms for nonnegative matrix factorization ［C］/ /Proceedings of Advances in Neural Information Processing Systems，2001: 556 － 562
